# Concerning trends in allopathic medical school faculty rank for Indigenous people: 2014–2016

**DOI:** 10.1080/10872981.2018.1508267

**Published:** 2018-08-14

**Authors:** Erik Brodt, Amanda Bruegl, Erin K. Thayer, M. Patrice Eiff, Kelly Gonzales, Carlos Crespo, Dove Spector, Martina Kamaka, Dee-Ann Carpenter, Patricia A. Carney

**Affiliations:** a Department of Family Medicine, Oregon Health & Science University, Portland, OR, USA; b Department of Obstetrics & Gynecology, Oregon Health & Science University, Portland, OR, USA; c Department of Family Medicine, Oregon Health & Science University, Portland, OR, USA; d Community Health Program, School of Public Health, Oregon Health & Science and Portland State University, Portland, OR, USA; e Community Health Program, Undergraduate Training Biomedical Research, Oregon Health & Science University and Portland State University, School of Public Health, Portland, OR, USA; f Department of Native Hawaiian Health, University of Hawaii, John A. Burns School of Medicine, Honolulu, HI, USA

**Keywords:** Medical school faculty, faculty rank, race/ethnicity, Indigenous people, diversity

## Abstract

**Background:** Trends in faculty rank according to racial and ethnic composition have not been reviewed in over a decade.

**Objective:** To study trends in faculty rank according to racial and ethnicity with a specific focus on Indigenous faculty, which has been understudied.

**Methods:** Data from the Association of American Medical Colleges’ Faculty Administrative Management Online User System was used to study trends in race/ethnicity faculty composition and rank between 2014 and 2016, which included information on 481,753 faculty members from 141 US allopathic medical schools.

**Results:** The majority of medical school faculty were White, 62.4% (*n* = 300,642). Asian composition represented 14.7% (*n* = 70,647). Hispanic, Latino, or of Spanish Origin; Multiple Race-Hispanic; Multiple Race-Non-Hispanic; and Black/African American faculty represented 2.2%, 2.3%, 3.0%, and 3.0%, respectively. Indigenous faculty members, defined as American Indian/Alaska Native (AIAN), Native Hawaiian or Other Pacific Islander (NHPI), represented the smallest percentage of faculty at 0.11% and 0.18%, respectively. White faculty predominated the full professor rank at 27.5% in 2016 with a slight decrease between 2014 and 2016. Indigenous faculty represented the lowest percent of full professor faculty at 5.2% in 2016 for AIAN faculty and a decline from 4.6% to 1.6% between 2014 and 2016 for NHPI faculty (p < 0.001).

**Conclusions:** While US medical school faculty are becoming more racially and ethnically diverse, representation of AIAN faculty is not improving and is decreasing significantly among NHPI faculty. Little progress has been made in eliminating health disparities among Indigenous people. Diversifying the medical workforce could better meet the needs of communities that historically and currently experience a disproportionate disease burden.

## Introduction

Approximately 15 years ago, several published papers characterized the racial and ethnic composition and faculty rank in US allopathic medical schools [1–5]. These papers collectively reported that White non-Hispanic race was the most prevalent majority composing 81–82% of all faculty. Underrepresented minorities (URM) and non-underrepresented minorities (NURM, such as Asian or Middle Eastern descent) occupied only 10% and 8% of the faculty positions, respectively [1–3]. Among URM faculty, 8.5% were reported as Black and 3.0% as Hispanic, while Asians (NURM) were reported as 7.5% [1]. This study did not include Indigenous faculty, including American Indians/Alaska Natives (AIAN), and Native Hawaiians or Other Pacific Islanders (NHPI). Over the past 15 years the understanding that a diverse medical workforce is necessary for health equity has grown [6]. However, there continues to be a paucity of published research that describes the racial and ethnic composition of faculty within US medical schools according to rank. These data are particularly lacking with regard to Indigenous faculty.

Reports on the impact of racial/ethnic distribution on faculty rank are conflicting. Peterson et al. [3] found that 48% of URM and 26% of NURM perceived racial/ethnic bias in their academic environment and had lower levels of career satisfaction. However, these same faculty members received comparable salaries, published equivalent numbers of papers, and were similarly likely to have attained senior rank (full or associate professor) [3]. In contrast, another study [2] found that White faculty had significantly more first-authored and total peer-reviewed publications than minority faculty groups. After adjustments for medical school, department, years as medical school faculty, number of peer-reviewed publications, receipt of research grant funding, the proportion of time spent on clinical activities, gender, and tenure status, this study found that the odds of holding senior rank were lower for Black faculty when compared to the odds of holding senior rank for White faculty (0.33; 95% confidence interval [CI], 0.17–0.63) [1].

Other studies into these issues have identified barriers that may inhibit advancement in rank among URM faculty. For example, URMs are more likely than Whites to have fewer and inadequate educational opportunities to prepare them for certain careers [5]. In turn, inadequate educational opportunities may undermine URMs’ ability to gain qualifications necessary for training programs and faculty positions [5]. Additionally, academic institutions often lack systematic and culturally relevant resources, including mentorship, to bolster retention among URM faculty and students [4]. Such resources are needed to overcome disparities in graduation rates, as well as institutional racism and implicit bias that may limit hiring and promotion processes among URMs.

As the US population continues to become more diverse, studies on advancement among minority faculty are important because an analogous physician workforce may improve racial and ethnic health disparities, including patient engagement [7], and delivery of culturally appropriate and responsive healthcare [8,9]. Indigenous people lag behind the general population on many socioeconomic factors and experience higher rates of morbidity and mortality compared to other racial and ethnic groups [8–12]. Factors that may contribute to these disparities include higher rates of unmet healthcare need and lower rates of healthcare use even when accounting for medical access [13]. Other inequities such as perceived experiences of discrimination occur not only in everyday settings [14], but also within the healthcare system itself [15–17]. These experiences contribute to disengagement in healthcare processes, lower levels of activation, and poorer health outcomes for Indigenous people [17].

Despite the promising benefits of an analogous medical workforce and the disproportionate health burdens experienced by Indigenous peoples, the number of AIAN US medical school applicants has actually declined by 32% between 1980 and 2013 [18]. Moreover, this negative trend is accelerating with a 70% decline in AIAN applicants and 63% decline in AIAN matriculants to US medical schools between 1996 and 2016 [18]. Nationally, NHPI matriculation to medical school has decreased by 46% over the past 4 years [18]. However, data from the John A. Burns School of Medicine (JABSOM) at the University of Hawaii, which accounts for the majority of NHPI medical school matriculation, shows NHPI matriculation has increased from an average of 6% in each class from 2003 through 2014 to an average of about 13% during the past 4 years [19]. Although increasing, these numbers remain well below what is needed for parity in the NHPI community. These findings suggest that efforts to diversify the medical workforce are lacking, especially for Indigenous peoples. An inadequate pool of AIANs and NHPIs matriculating into medical school directly leads to an inadequate pool of medical school faculty.

The goal of these analyses was to reexamine recent trends in faculty rank according to race and ethnicity among US medical school faculty to assess the status of URM academic career advancement relative to its majority counterpart. Because data have only recently been available on Indigenous faculty, understanding trends in this URM group is a specific focus of this work.

## Methods

We used reports produced from the Association of American Medical Colleges (AAMC) Faculty Administrative Management Online User System (FAMOUS) to create a dataset to study trends in faculty rank according to race/ethnicity between 2014 and 2016. The AAMC defines URM as those racial and ethnic populations that are underrepresented in the medical profession relative to their numbers in the general population [20]. Medical schools routinely upload faculty roster information to FAMOUS, a web-based database that launched in 2002 and houses data on faculty retention, promotion, alumni, educational background, training, departmental affiliations, and demographic reports. Beginning in 2009, the LCME began using the faculty roster data to reflect each medical school’s full-time faculty counts. Thus, it is fairly representative of allopathic medical school faculty.

More specifically, we used data from the demographic and promotion reports to create the dataset for these analyses by writing macros in Excel that would populate a spreadsheet with data that matched those provided in the report for each year under study (2014, 2015, and 2016). Variables included race, ethnicity and faculty rank on 481,753 faculty members from 141 allopathic US medical schools. We then uploaded the data into SPSS version 23 for analyses. The data in FAMOUS are static, and to reduce the possibility of incomplete data, we allowed 9 months to pass before examining trends between 2014 and 2016 to allow 2016 data to be as complete as possible.

We used descriptive statistics to characterize race/ethnicity data according to calendar year and conducted a trend analysis on changes in faculty rank according to race and ethnicity with alpha level for statistical significance set at 0.05. Our results are provided in aggregate and not stratified by gender. We know from published literature that between 2014 and 2016, the distribution of male and female faculty in medical school did not change substantially [21,22]. Oregon Health & Science University’s Institutional Review Board reviewed and determined this study to be exempt as it does not actively involve human subjects (IRB#17688). To provide additional context for the data presented, we added US Census data from 2016 [23] and available data from a paper published in JAMA in 1998 [1] to Table 1. Of note is that the JAMA paper represented a stratified random sample of full-time faculty from 24 representative US medical schools while data set we analyzed included the faculty rosters from all medical schools.

**Table 1.** Medical school faculty race/ethnicity by year compared to national population averages from 2016.

US allopathic physician faculty population					July 2016 US population	
Race/ethnicity	1995^a^ *n* (%)	2014 *n* (%)	2015 *n* (%)	2016 *n* (%)	Totals *n* (%)	*n* (%)
White	1463 (80.9%)	96,696 (62.3%)	100,750 (63.0%)	103,196 (61.9%)	300,642 (62.4%)	248,485,058 (76.9%)
Multiple race – Hispanic	–	3692 (2.4%)	3753 (2.4%)	3847 (2.3%)	11,292 (2.3%)	–
Multiple Race, ethnicity not specified	–	–	–	–	–	8,401,315 (2.6%)
Hispanic, Latino, or of Spanish Origin	54 (3.0%)	3001 (1.9%)	3439 (2.2%)	4195 (2.5%)	10,635 (2.2%)	57,516,697 (17.8%)
Multiple race – non-Hispanic		4547 (2.9%)	4838 (3.0%)	5274 (3.2%)	14,659 (3.0%)	–
Asian	136 (7.5%)	21,730 (14.0%)	23,317 (14.6%)	25,600 (15.4%)	70,647 (14.7%)	18,418,268 (5.7%)
Black/African American	154 (8.6%)	4514 (2.9%)	4799 (3.0%)	5075 (3.0%)	14,388 (3.0%)	42,975,959 (13.3%)
American Indian/Alaska native	–	173 (0.11%)	186 (0.11%)	194 (0.12%)	553 (0.11%)	4,200,658 (1.3%)
Native Hawaiian/Pacific Islander	–	392 (0.25%)	285 (0.17%)	184 (0.11%)	861 (0.18%)	646,255 (0.2%)
American Indian/Alaska Native/Native Hawaiian/Pacific Islander combined	–	565 (0.36%)	471 (0.28%)	278 (0.23%)	1414 (0.29%)	4,846,913 (1.5%)
Unknown	–	19,733 (12.7%)	17,703 (11.1%)	18,388 (11.0%)	55,824 (11.6%)	–
Total	1808	155,209	159,831	166,713	481,753	323,127,513

^a^ Reported in *JAMA*: Palepu A, Carr PL, Friedman RH, Amos H, Ash AS, Moskowitz MA. Minority Faculty and Academic Rank in Medicine. JAMA, 1998;280(9):767–771

## Results

**Figure 1.**
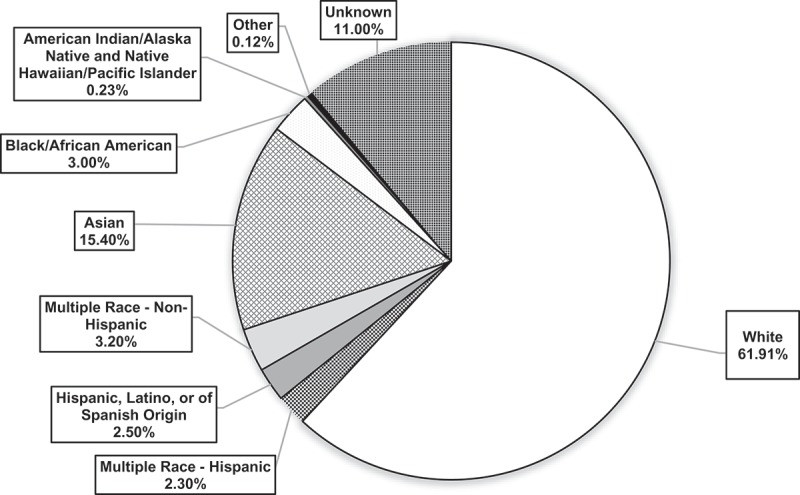
Race ethnicity breakdown in 2016 (*N* = 166,713).

Non-Hispanic White individuals represented 62.4% (*n* = 300,642) of the faculty, and Asian faculty members were second most common at 14.7% (*n* = 70,647) during the study period (Table 1). Faculty who were Hispanic, Latino, or of Spanish Origin; Multiple Race-Hispanic; Multiple Race-Non-Hispanic; and Black/African American represented 2.2%, 2.3%, 3.0%, and 3.0%, respectively. Indigenous Physicians, MD, and DO, comprise 0.36% of all physicians in the USA [24] (*data not shown*). Indigenous faculty members, AIANs and NHPIs, represented the smallest proportion of medical school faculty at 0.11% and 0.18%, respectively, with the percent of Indigenous faculty (both categories combined) representing 0.29%. Regarding doctoral training in other disciplines, the total number of AIAN PhDs was 5600 (0.79%) and NHPIs was 1500 (0.21%) in 2008 [25] (*data not shown*). In addition, the US population of AIANs is 1.5%; thus, the proportion of AIANs in medicine is less than that of the population. Also shown in Table 1 is the available race and ethnic background of allopathic physicians from a study conducted in 1995 [1]. This study, which was based on a stratified random sample of full-time faculty from 24 representative US medical schools, indicated that White faculty represented 80.9% of all allopathic medical school faculty, though at that time very few racial categories were collected. In contract in 2016, White representation among medical school faculty was lower at 62.4% than occurred in 1995. In addition, it is lower than its representation in the general population at 76.9%, which is also shown in Table 1, according to the US Census [11]. Figure 1 illustrates the overall distribution of faculty in the most current year (2016), showing that AIAN and NHPI categories combined made up 0.23% of faculty in 2016.

The proportion of faculty who were Non-Hispanic White, Black/African American, Multiple Race-Hispanic, and AIAN remained fairly stable between 2014 and 2016, while slight increases were noted among faculty who were Hispanic, Latino, or of Spanish Origin, Multiple Race-Non-Hispanic or AIAN. Faculty who were NHPI were the only group that decreased steadily between 2014 and 2016.

**Figure 2.**
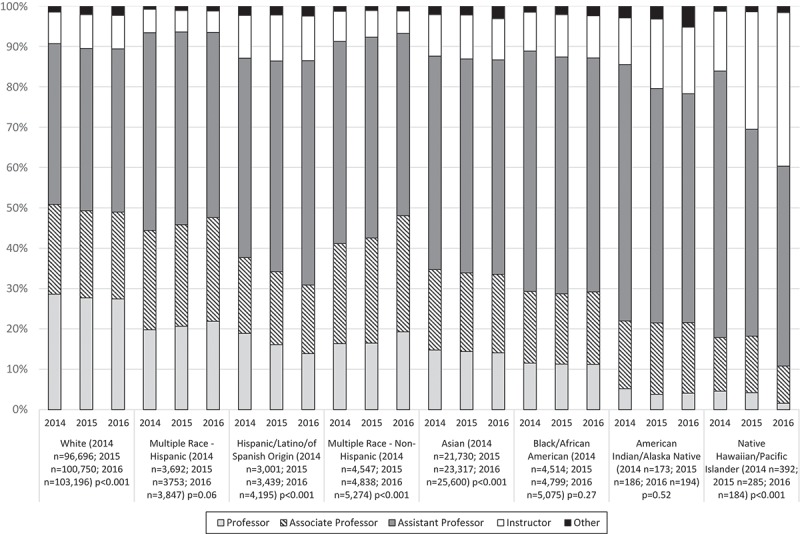
Medical school facility rank according to race/ethnicity 2014–2016.

Trends in faculty rank between 2014 and 2016 according to race/ethnicity are shown in Figure 2. For professor rank, Whites had the highest percentage of this rank at 28.6% in 2014, with a slight though statistically significant reduction to 27.5% in 2016 (*p* < 0.001). Multi-race-Hispanic was the second highest group at the professor rank with slight increases over time at 19.8% in 2014 to 21.9% in 2016 (*p* = 0.06). Indigenous faculty represented the lowest percent of professor faculty at 3.8% in 2014, with a slight increase to 5.2% in 2016 for AIAN faculty and statistically significant decline from 4.6% in 2014 to 1.6% in 2016 (*p* < 0.001) for NHPI faculty with a corresponding increase in the proportion of instructors/assistant professors.

## Discussion

Findings from this study confirm that representation of Indigenous faculty at US allopathic medical schools is abysmal. Over a decade ago, the vast majority of medical school faculty were White (>80%). We found that this percentage declined by approximately 20%, which indicates that White representation is now lower than it is in the general population at 76.9% [23]. We found mixed trends over time for URM faculty. Representation has increased for some groups (Hispanic and Non-Hispanic Multi-race faculty and Asian faculty), decreased for some (NH), and remained relatively stable for others (Black/African American and AIAN). Clearly, efforts are needed to recruit, retain, and diversify the faculty workforce. For example, studies have shown that racially and ethnically congruent mentor-URM student relationships may positively contribute to the medical school training experience and student progress [26]. This is especially important as coaching models are increasingly being implemented in medical schools, and at least one study has found that URM medical students have different coaching needs based on their cultures compared to NURM medical students. In this study, focus groups were conducted with URM and NURM medical students in their second or third year of training, which found that URM students experienced significant stress about sharing fears of possible harms or vulnerabilities related to their race with faculty from majority race/ethnicity, a finding that did not exist among NURM students (Carney PA. Personal Communication (07/07/2018)).

Several studies of medical school faculty have been conducted [27–29], one of which revealed that 34% of new hires resigned within 3 years [27]. Among these faculty, reasons for leaving included issues with inclusiveness, respect, open communication, lack of professional development of the faculty member, and being overwhelmed by clinical care. This study, which was conducted in 2014, found that gender, race, ethnicity, academic degree, department type, and tenure status did not predict early attrition [27]. Two other studies highlighted challenges for academic advancement, although they did not include data on race or ethnicity. A 2007 study of 1408 faculty members [28] found that 40% perceived their careers were not progressing satisfactorily and 42% were considering leaving academic medicine. The second study looked at gender differences [29] and found that compared to men, female faculty reported a lower sense of belonging, lower self-efficacy for career advancement and a lower perception that their institutions were making changes to address diversity goals.

Training more Indigenous people does have the potential to alleviate current workforce challenges that exist throughout Indian country and NHPI communities because Indigenous health professionals are more likely to serve Indigenous communities compared to their peers [8], which could have a significant impact on AIAN and NHPI health. AIANs face some of the most striking health inequities in the USA, living with the highest rates of diabetes and heart, lung, and blood disease, and they die younger than any other racial or ethnic group [30,31]. National Data on Native Hawaiians (NH) is frequently aggregated with those of Asians and/or Pacific Islanders making it difficult to look at NH-specific statistics. Nationally, NHPIs suffer from higher prevalence of CVD and Diabetes compared to the US average [32]. When looking at State of Hawaii data, where 55% of NH live [33], NH suffer from the highest prevalence and mortality rates of cardiovascular disease, diabetes, breast cancer, and lung cancer as well as the lowest life expectancy of all major ethnic groups [34–37].

The scarcity of Indigenous faculty raises several issues. First, are Indigenous faculty being recruited to remain in academic institutions after completion of training? Second, are academic institutions maximizing efforts to ensure the success of URM faculty groups in terms of promotion and tenure? Third, is cultural humility a valued element of the academic environment? Finally, does the rarity of Indigenous faculty limit opportunities for young Indigenous students to access physician and scientist role models along their educational pathways? One critical step toward decreasing these inequities would be to train and support the academic success of Indigenous physicians in general, and then fostering further opportunities to develop some of these Indigenous physicians into faculty members. The Northwest Native American Center of Excellence is an example of a comprehensive program designed to increase the number of AIANs in medicine by providing enrichment across the educational continuum, beginning with Indigenous youth up to Indigenous faculty.

One explanation of the paucity of Indigenous graduates from US Medical schools is negatively impacting the applicant pool of Indigenous faculty. Without graduating Indigenous physicians from US Medical Schools, there will not be an applicant pool for academic faculty. Hollow et al. reported the concordance of AIAN faculty-student relationships on the success of AIAN medical students throughout their educational pathway [38]. The *Diversity in Medical Education: Facts and Figures 2016* report from the AAMC highlights the abysmal shortage of Indigenous graduates, twenty [20] AIAN and five [5] NHPI medical school graduates in 2015 [39].

These data report AIAN or NHPI ‘alone’ self-identification, where respondents indicating multiple races are counted separately. It may be that AIAN and NHPIs fall into this category. In any case, there is a need to increase the number of highly competitive Indigenous applicants to US medical schools as well as identify creative solutions to increase the number of US Medical Schools graduating Indigenous physicians. Our center is currently participating in a report with the AAMC and the Association of American Indian Physicians to highlight best practice models for AIANs in the medical education pathway. An additional explanation for the low numbers of Indigenous faculty and the decline among NHPI faculty found in this study may be that students are not seeing enough role models in either academic medical centers or in health clinics, which might adversely affect their decision to pursue a medical career.

One approach to improve recruitment could involve centralizing recruitment efforts to reduce variability, improve the use of tangible resources, and facilitate sharing of best recruitment practices across departments [40]. Academic medical centers should develop multifaceted strategies to address the challenges faced by Indigenous faculty. These challenges include limited information on academic career paths, lack of credit for teaching and community service, isolation, challenges in balancing Indigenous and academic cultures, and lack of role models/mentors [41]. Additionally, Indigenous faculty, like other URM faculty, experience the ‘minority tax’ and are burdened with increased institutional service commitments to help with diversity efforts, racism, and awareness of inequities [42]. Faculty development programs could also do a better job of specifically addressing the career advancement needs of Indigenous faculty. For example, few development opportunities exist that specifically support the nuances of conducting tribal research, navigating Indigenous cultural and academic responsibilities, and isolation in academia for Indigenous faculty. Both the Northwest Native American Center of Excellence (NNACOE) and the Native Hawaiian Center of Excellence (NHCOE) have begun to address these issues by providing Indigenous Faculty Forums or Fellowships, which includes attending the JABSOM Fellowship in Medical Education as well as opportunities to present at national Indigenous conferences. We will be closely studying the impact of such programs over time.

Much more research is needed to address the commitment of US medical schools to reduce health disparities. We need to determine why Indigenous people with medical or science training are not pursuing academic careers and then determine where they are going. Do cultural differences prevent Indigenous people with advanced training from being viewed as academically proficient by senior faculty or institutional leaders? Do Indigenous applicants have the same access or equal opportunity to compete for academic positions compared to other racial/ethnic groups? We need more research to understand if Indigenous people are not well prepared during their training to become academicians, which might explain the lack of equal access.

A strength of this study is the large dataset we were able to create using reports from the AAMCs FAMOUS dataset, which contained race/ethnicity classification for the majority of the 481,753 faculty members included in the file between 2014 and 2016. Limitations also exist, which include the possibility that this static dataset may not be complete, though we did undertake a 9-month waiting period after the end of 2016 before pulling data for these analyses. Another weakness is that there may be misclassification errors where AIAN, NHPI were attributed to mixed race or other categories by those submitting data to FAMOUS. We did not include sex of faculty as our primary focus was on differences in trends among URM versus NURM and White faculty between 2014 and 2016. Had we included sex, our findings for faculty rank may have been even more informative; however, the gender distribution of faculty in allopathic schools has not changed substantially from 2014 to 2016. Lastly, 58,070 faculty members submitted to the FAMOUS site were considered either ‘Other’ (0.47%) or ‘Unknown’ (11.6%), representing about 12.1% of the dataset. This is not an insignificant amount of missing data. If the Unknown category was smaller, we would worry less about misclassification error. It is unlikely that there would be a large number of Indigenous faculty selecting other or unknown.

While faculty at US medical schools are becoming more diverse with respect to race/ethnicity, the representation of Indigenous people is not improving for AIAN faculty and is decreasing significantly among NHPI faculty. These trends must be reversed to assist in improving health outcomes of Indigenous people, as a non-diverse faculty may limit innovative solutions to improve the health of Indigenous people. More research is needed on how best to support recruitment and academic progression efforts among Indigenous faculty in academic medical centers.
